# Genetic causes of Parkinson’s disease in the Maltese: a study of selected mutations in *LRRK2*, *MTHFR*, *QDPR* and *SPR*

**DOI:** 10.1186/s12881-016-0327-x

**Published:** 2016-09-09

**Authors:** Charmaine Zahra, Christine Tabone, Graziella Camilleri, Alex E. Felice, Rosienne Farrugia, Stephanie Bezzina Wettinger

**Affiliations:** 1 Laboratory of Molecular Genetics, Department of Physiology and Biochemistry, University of Malta, Msida, Malta; 2 Department of Applied Biomedical Science, Faculty of Health Sciences, University of Malta, Msida, Malta

**Keywords:** Quinoid Dihydropteridine Reductase, Sepiapterin Reductase, Methylenetetrahydrofolate Reductase, Leucine-rich repeat kinase 2, Alpha-synuclein, Parkinson’s Disease, Maltese

## Abstract

**Background:**

Mutations in Leucine-rich repeat kinase 2 NM_198578 (*LRRK2* c.6055G > A (p.G2019S), *LRRK2* c.4321C > G (p.R1441G)) and alpha-synuclein NM_000345 (*SNCA* c.209G > A (p.A53T)) genes causing Parkinson’s disease (PD) are common in Mediterranean populations. Variants in the Quinoid Dihydropteridine Reductase NM_000320 (*QDPR* c.68G > A (p.G23D)), Sepiapterin Reductase NM_003124 (*SPR* c.596-2A > G) and Methylenetetrahydrofolate Reductase NM_005957 (*MTHFR* c.677C > T and c.1298A > C) genes are frequent in Malta and potential candidates for PD.

**Methods:**

178 cases and 402 control samples from Malta collected as part of the Geoparkinson project were genotyped for *MTHFR* polymorphisms, *QDPR* and *SPR* mutations. Only PD and parkinsonism cases were tested for *SNCA* and *LRRK2* mutations.

**Results:**

*LRRK2* c.4321C > G and *SNCA* c.209G > A were not detected. The *LRRK2* c.6055G > A mutation was found in 3.1 % of Maltese PD cases. The *QDPR* mutation was found in both cases and controls and did not increase risk for PD. The *SPR* mutation was found in controls only. The odds ratios for *MTHFR* polymorphisms were not elevated.

**Conclusions:**

The *LRRK2* c.6055G > A is a cause of PD in the Maltese, whilst *QDPR* c.68G > A, *SPR* c.596-2A > G and *MTHFR* c.677C > T and c.1298A > C are not important determinants of PD.

**Electronic supplementary material:**

The online version of this article (doi:10.1186/s12881-016-0327-x) contains supplementary material, which is available to authorized users.

## Background

Parkinson’s disease (PD) is a neurodegenerative disorder characterised by motor and non-motor symptoms and loss of dopaminergic neurons. A number of candidate genes were investigated to uncover the PD causing mutations in the Maltese population. Mutations in leucine-rich repeat kinase 2 NM_198578 (*LRRK2*) and alpha-synuclein NM_000345 (*SNCA*) genes have been associated with PD. Three mutations in these genes, namely *LRRK2* c.6055G > A, *LRRK2* c.4321C > G and *SNCA* c.209G > A are pathogenic mutations found in Mediterranean populations [1]. The Quinoid Dihydropteridine Reductase NM_000320 (*QDPR*) c.68G > A mutation and Sepiapterin Reductase NM_003124 (*SPR*) c.596-2A > G splice variant, are autosomal recessive mutations known to cause dihydropteridine reductase (DHPR) deficiency and Dopa-Responsive Dystonia (DRD) respectively in the Maltese with a high carrier frequency compared to other populations [2]. Catecholamine synthesis in patients is impaired as tetrahydrobiopterin (BH_4_), an essential cofactor of aromatic amino acid hydroxylases, is not produced in sufficient amounts as a result of these mutations [3]. SR is involved in *de novo* BH_4_ synthesis whilst DHPR is involved in recycling of the reduced cofactor [4]. It has been suggested that the *SPR* gene is the PARK3 locus implicated in sporadic PD [1] and decreased activity of DHPR due to low biopterin levels causes PD [5]. DHPR deficiency and DRD and PD have symptoms in common. The latter is characterized by childhood-onset dystonia responding well to low doses of L-Dopa. Gait and posture abnormalities are observed as the legs are primarily affected with diurnal fluctuation. Symptoms of parkinsonism (PS) are common. DHPR deficiency also presents with tonic disorders, abnormal movements, psychomotor retardation, hypersalivation and drooling, swallowing difficulties, hyperthermia [3, 6]. There is evidence that both the *SPR* and *QDPR* genes are good candidates to test for associations for PD.

MTHFR reduces folate and is involved in the production of S-adenosylmethionine which plays a role in dopamine (DA) synthesis and metabolism [7]. Defective MTHFR enzyme due to *MTHFR* NM_005957 c.1298A > C and c.677C > T has a lower activity and may result in elevated homocysteine levels, found to be relatively high in sera of PD patients [7, 8]. An indirect connection between the BH_4_ and folate pathways has been postulated [9]. The *MTHFR* c.677C > T TT genotype is found at a frequency of 9.1 % in Maltese newborn [10]. The mutant allele frequency for the *MTHFR* c.1298A > C was reported to be 23.2 % [11].

The objective of this study was to establish whether known pathogenic mutations (*LRRK2* c.6055G > A, *LRRK2* c.4321C > G and *SNCA* c.209G > A) are present in Maltese PD and PS cases, and to determine whether the functional *MTHFR* c.677C > T and c.1298A > C, *QDPR* c.68G > A and *SPR* c.596-2A > G genetic variants alter susceptibility to PD. This is the first time these genes have been tested for involvement in PD in the Maltese population.

## Methods

Altogether 178 cases and 402 controls (Table 1) from Malta were collected as part of the FP5 EU-funded Geoparkinson project [12, 13]. The collection and testing were approved by the Research Ethics Committee of the University of Malta, Approval Numbers 53/2002 and 52/2008. All participants gave written informed consent.

**Table 1:** Characteristics of the population studied

Characteristics	Cases (*N =* 178)	Controls (*N =* 402)
Mean age (range)	72.9 (41–91)	74.3 (41–95)
Mean age of diagnosis^a^	66.3 (35–90)	
Males	110 (61.8 %)	224 (55.7 %)
Females	68 (39.2 %)	178 (44.3 %)
Parkinson’s Disease	118	
Parkinsonism	60	
% Parkinsonism	33.70 %	
Smokers^d^	61 (34.3 %)	177 (44.0 %)
Ever knocked unconscious^e^	33^b^ (18.6 %)	48 (11.9 %)
Family history of Parkinson’s disease^e^	35 (19.7 %)	16^c^ (4.0 %)
Use of pesticides^e^	27 (15.2 %)	42 (10.4 %)

^a^Out of 135 cases for which this data was available

^b^Out of 177 cases

^c^Out of 401 controls

^d^Associated with decreased risk for PD and PS

^e^Associated with increased risk for PD and PS

Cases were classified as having PD or PS using the United Kingdom Parkinson’s Disease Society Brain Bank clinical diagnostic criteria. Controls did not have PD or PS and were recruited from the community and from the bleeding room at St Luke’s Hospital which served many outpatient departments.

Genotyping of the *QDPR* c.68G > A, *SPR* c.596-2A > G [2], *MTHFR* c.677C > T [14], *LRRK2* c.6055G > A [15], *LRRK2* c.4321C > G [16] and *SNCA* c.209G > A [17] was performed as described previously. Genotyping of the *MTHFR* c.1298A > C was done using the 5’ exonuclease assay [18]. Cases and controls were genotyped for the *QDPR* c.68G > A, *SPR* c.596-2A > G mutations and the *MTHFR* c.677C > T and c.1298A > C polymorphisms. Genotyping for the *LRRK2* c.6055G > A, *LRRK2* c.4321C > G and *SNCA* c.209G > A was limited to cases since these are known PD-causing mutations [1].

DNA of 79 controls and 27 cases collected as part of the Geoparkinson project had been used up. These were mainly buccal samples. Additionally the PCRs did not work in a few samples.

Data Analysis: Allele frequencies were calculated. The genotypes in controls, when available, were tested for conformity with the Hardy-Weinberg Equilibrium (HWE). Both the crude and adjusted odds ratios (ORs) were calculated and the 95 % confidence intervals (CI) were derived from the logistic regression model.

## Results

Four cases were heterozygous for the *LRRK2* c.6055G > A mutation, giving an allele frequency of 1.4 % in PD and PS cases. This frequency was 3.1 % in PD cases alone. All were males with no family history of disease. Data for age at diagnosis was only available for two cases, one with PD and one with PS, both diagnosed at 65 years of age. None of the cases had the *LRRK2* c.4321C > G or *SNCA* c.209G > A mutations. The frequencies obtained for the variants studied are presented in Tables 2 and 3.

**Table 2:** Allele Frequencies for the variants studied

Mutation/polymorphism	Allele frequencies in %	
	Controls (n mutant/total n)^a^	Cases (n mutant/total n)^a^
*QDPR* c.68G > A	0.3 (2/612)	0.3 (1/300)
*SPR* c.596-2A > G	0.7 (4/594)	0.0 (0/296)
*MTHFR* c.677C > T	34.7 (216/622)	33.8 (102/302)
*MTHFR* c.1298A > C	35.9 (225/626)	37.7 (114/302)
*Tested in cases only*		
*LRRK2* c.6055G > A	-	1.4 (4/292)
*LRRK2* c.4321C > G	-	0.0 (0/296)
*SNCA* c.209G > A	-	0.0 (0/292)

*n* = number of alleles

^a^DNA of 79 controls and 27 cases had been used up and were therefore not available and a few samples did not yield PCR products

**Table 3:** Genotype Frequencies for MTHFR, all cases (PD & PS)

Polymorphism		Genotype frequencies			*p-*value
*MTHFR* c.677C > T	*MTHFR* c.1298A > C	No. of Cases (%)^a^	No. of Controls (%)^a^	Age-adjusted OR (95 % CI)^b^	
CC		66 (43.7)	136 (43.7)	1	
CT		68 (45.0)	134 (43.1)	1.0 (0.7–1.6)	0.8
TT		17 (11.3)	41 (13.2)	0.9 (0.5–1.6)	0.6
	AA	60 (39.7)	132 (42.2)	1	
	AC	68 (45.0)	137 (43.8)	1.1 (0.7–1.7)	0.7
	CC	23 (15.2)	44 (14.1)	1.2 (0.6–2.1)	0.6
CC	AA	16 (10.6)	24 (7.7)	1	
CT	AC	41 (27.2)	67 (21.6)	0.9 (0.4–2.0)	0.8
TT	CC	-	-	-	
CC	AC	27 (17.9)	69 (22.3)	0.6 (0.3–1.3)	0.2
CC	CC	23 (15.2)	43 (13.9)	0.8 (0.4–1.8)	0.6
CT	AA	27 (17.9)	66 (21.3)	0.6 (0.3–1.3)	0.2
CT	CC	-	-	-	
TT	AA	17 (11.3)	41 (13.2)	0.6 (0.3–1.5)	0.3
TT	AC	-	-	-	

^a^DNA of 79 controls and 27 cases had been used up and were therefore not available and a few samples did not yield PCR products

^b^ *p-values were all >0.05*

Odds ratios for combined PD and PS cases were OR 0.9 (95 % CI 0.5–1.6) for the *MTHFR* 677 TT and 1.2 (95 % CI 0.6–2.1) for the *MTHFR* 1298 CC genotypes respectively. The results did not change materially after restricting to cases with PD or PS (see Additional file 1). No deviations from HWE were observed.

The allele frequency for *QDPR* c.68G > A was 0.3 % in both cases and controls. Two controls and one case were heterozygous for *QDPR* c.68G > A. One control was a 73 year old male whilst the other control was a 66 year old female. The case identified fulfilled the criteria for PD. She was 53 years of age when diagnosed and was also heterozygous for the *MTHFR* c.1298A > C polymorphism. None had a family history of PD.

Four individuals were identified as carriers of *SPR* c.596-2A > G, all of which were controls giving an allele frequency of 0.7 %. One was female aged 70, whilst the others were all men with ages of 79, 49 and 66.

## Discussion

The known disease-causing *LRRK2* c.6055G > A mutation was detected in PD cases whereas the *LRRK2* c.4321C > G and *SNCA* c.209G > A mutations were not detected. The allele frequency of *QDPR* c.68G > A was the same in the cases and the controls whilst *SPR* c.596-2A > G was not detected in cases. The *MTHFR* c.677C > T and *MTHFR* c.1298A > C polymorphisms were not associated with PD in this study.

Four cases were heterozygous for the *LRRK2* c.6055G > A mutation giving an allele frequency of 1.4 % in PD and PS cases or 3.1 % in PD cases alone. *LRRK2* (c.4321C > G and c.6055G > A) mutations cause amino acid changes within the GTPase and/or kinase domains respectively which may signify an enzymatic change and thus mediate *LRRK2* pathogenesis [19, 20]. The frequency of *LRRK2* c.6055G > A was estimated to be 1 % of patients with sporadic PD and 4 % of patients with hereditary PD in other populations, with the highest frequencies occurring in Arabs and Jews but is rare in Asia [21]. This may be explained by differences in descent and founder effects [22]. The frequency of this mutation in Maltese PD patients, 3.1 %, is similar to that in the Spanish, Italians and Sardinians [21]. This is the first report of this mutation being found in the Maltese.

The *LRRK2* c.4321C > G is common in the Basque population [23, 24]. The *SNCA* c.209G > A mutation is thought to have arisen from a common founder with Mediterranean ancestry [25, 26]. The results of the present study indicate that the *LRRK2* c.4321C > G and *SNCA* c.209G > A mutations are very rare or completely absent in the Maltese population.

The allele frequencies for the *QDPR* c.68G > A and *SPR* c.596-2A > G we observed in controls (0.3 % and 0.7 % respectively) are much lower than those reported previously (3.3 % and 4.6 % tested on 272 and 87 newborns respectively) [2]. Therefore, we tested the population frequency in a larger number of Maltese newborn and observed allele frequencies of 0.64 %, and 1.3 % for the *QDPR* and *SPR* polymorphisms respectively (*N =* 475 and 315 respectively). These mutations are not a major cause of PD in the Maltese.

*MTHFR* was not a contributor to PD. The genotype frequencies obtained for the *MTHFR* c.677C > T are 43.7 % CC, 43.1 % CT and 13.2 % TT which are comparable to others reported for Maltese newborn [10, 27]. In contrast, the mutant allele frequency for the *MTHFR* c.1298A > C was somewhat higher than that observed previously in Maltese newborn (35.9 % vs. 23.2 % [11]). This suggests that there is no clear effect of these SNPs on PD probably due to the complex pathways involved.

The c.677C > T and c.1298A > C prevalence and frequencies vary considerably with ethnicities and geography [7]. While some studies have reported a lack of association between *MTHFR* polymorphisms and PD [28], others [29] reported a higher frequency of the *MTHFR* 677 TT genotype in PD patients and controls.

The *MTHFR* c.667C > T dimorphism causes hyperhomocysteimia in folate deficient individuals [30]. Homozygous mutants for *MTHFR* c.1298A > C have a lower enzyme activity than homozygous wildtype [7]. It is possible that the Maltese diet includes sufficient folate levels. In fact only 1.5 % of adult Maltese women and 3.1 % of adult Maltese men had low serum folate levels in a group of population controls and 1.5 % of women and 1.2 % of men had low serum Vitamin B12 levels (*N =* 137 and *N =* 326 for women and men respectively; Attard R and Bezzina Wettinger S, unpublished observations). The chance of being heterozygous or homozygous for these polymorphisms with low folate status is therefore very low. It should be noted that higher nutrient intake (folate and B vitamins) did not lower PD risk in a previous study [31]. It cannot be excluded that these polymorphisms have an effect in populations or individuals with low folate and Vitamin B12 levels.

This study had 80 % power to detect a 2-fold increase in risk for the *MTHFR* polymorphisms (35 % allele frequency), a 5-fold increased risk for *SPR* c.596-2A > G (0.7 % allele frequency) and an 8-fold increased risk for *QDPR* c.68G > A (0.3 % allele frequency) assuming a dominant model and for the *MTHFR* polymorphisms the sample size was sufficient to detect a two-fold increase in risk even for a recessive model assuming up to a 5 % Type I error rate. Thus the study is sufficiently powered to exclude a strong effect on PD risk of the *SPR* and *QDPR* mutations, and even 2-fold or more increase in risk due to the *MTHFR* mutations.

## Conclusion

The *LRRK2* c.6055G > A mutation accounts for 3.1 % of PD in the Maltese population. As *LRRK2* c.6055G > A exhibits low penetrance and is found occasionally in controls [21], testing for *LRRK2* c.6055G > A may be performed on patients and relatives who wish to ascertain the presence of a genetic defect. Testing for *LRRK2* c.4321C > G and *SNCA* c.209G > A are not recommended as a first line of genetic testing in Maltese PD patients. There is no evidence to support involvement of DHPR and SR in PD, at least alone. One cannot exclude that they have a weak effect and even possibly an interaction when found together with other mild mutations. The absence of the *LRRK2* c.4321C > G and *SNCA* c.209G > A could be a result of historical bottlenecks, and there may be some founder mutation in the Maltese that may explain familial cases that has not yet been tested for.

## Additional file

Additional file 1: Genotype frequencies for MTHFR, Parkinson's Disease (Table S1a) and Parkinsonism (Table S1b) only. (DOC 57.5 kb)

## Abbreviations

BH_4_: Tetrahydrobiopterin
CI: Confidence intervals
DA: Dopamine
DHPR: Dihydropteridine reductase
DRD: Dopa-Responsive Dystonia
HWE: Hardy-Weinberg Equilibrium
L-Dopa: Levodopa
LRRK2: Leucine-rich repeat kinase 2
MTHFR: Methylenetetrahydrofolate Reductase
OR: Odds ratios
PD: Parkinson’s disease
PS: Parkinsonism
QDPR: Quinoid Dihydropteridine Reductase
SNCA: Alpha-synuclein
SNP: Single Nucleotide Polymorphism
SPR: Sepiapterin Reductase
