# Supplementary material for: Genetic causes of Parkinson’s disease in the Maltese: a study of selected mutations in LRRK2, MTHFR, QDPR and SPR
Source: BMC Med Genet. 2016 Sep 9;17(1):65. doi: 10.1186/s12881-016-0327-x (PMC5016953; doi:10.1186/s12881-016-0327-x)
Supplement: Additional file 1: — Genotype frequencies for MTHFR, Parkinson's Disease (Table S1a) and Parkinsonism (Table S1b) only. (DOC 57.5 kb) [file 12881_2016_327_MOESM1_ESM.doc]

**Supplementary material**

**Table S1a:** Genotype frequencies for MTHFR, PD only

| **Polymorphism** | | **Genotype frequencies** | | | **p-value** |
| --- | --- | --- | --- | --- | --- |
| ***MTHFR*** c.677C>T | ***MTHFR*** c.1298A>C | **No. of Cases**  **(%)** | **No. of Controls (%)** | **Age-adjusted OR (95% CI)*** |  |
| CC |  | 40 (40.0) | 136 (43.7) | 1 |  |
| CT |  | 50 (50.0) | 134 (43.1) | 1.3 (0.8-2.1) | 0.3 |
| TT |  | 10 (10.0) | 41 (13.2) | 0.8 (0.4-1.8) | 0.6 |
|  | AA | 35 (35.0) | 132 (42.2 | 1 |  |
|  | AC | 52 (52.0) | 137 (43.8) | 1.4 (0.9-2.3) | 0.2 |
|  | CC | 13 (13.0) | 44 (14.1) | 1.1 (0.5-2.2) | 0.8 |
| CC | AA | 8 (8.0) | 24 (7.7) | 1 |  |
| CT | AC | 33 (33.0) | 67 (21.6) | 1.4 (0.6-3.6) | 0.4 |
| TT | CC | - | - | - |  |
| CC | AC | 19 (19.0) | 69 (22.3) | 0.8 (0.3-2.1) | 0.4 |
| CC | CC | 13 (13.0) | 43 (13.9) | 0.9 (0.3-2.4) | 0.8 |
| CT | AA | 17 (17.0) | 66 (21.3) | 0.8 (0.3-2.0) | 0.6 |
| CT | CC | - | - | - |  |
| TT | AA | 10 (10.0) | 41 (13.2) | 0.7 (0.2-2.0) | 0.5 |
| TT | AC | - | - | - |  |

**p-values were all >0.05*

**Table S1b:** Genotype frequencies for MTHFR, PS only

| **Polymorphism** | | **Genotype frequencies** | | | **p-value** |
| --- | --- | --- | --- | --- | --- |
| ***MTHFR*** c.677C>T | ***MTHFR*** c.1298A>C | **No. of Cases**  **(%)** | **No. of Controls (%)** | **Age-adjusted OR (95% CI)*** |  |
| CC |  | 26 (51.0) | 136 (43.7) | 1 |  |
| CT |  | 18 (35.3) | 134 (43.1) | 0.7 (0.4-1.4) | 0.3 |
| TT |  | 7 (13.7) | 41 (13.2) | 1.0 (0.4-2.4) | 0.9 |
|  | AA | 25 (49.0) | 132 (42.2) | 1 |  |
|  | AC | 16 (31.4) | 137 (43.8) | 0.6 (0.3-1.2) | 0.2 |
|  | CC | 10 (19.6) | 44 (14.1) | 1.2 (0.5-2.8) | 0.6 |
| CC | AA | 8 (15.7) | 24 (7.7) | 1 |  |
| CT | AC | 8 (15.7) | 67 (21.6) | 0.4 (0.1-1.2) | 0.1 |
| TT | CC | - | - | - |  |
| CC | AC | 8 (15.7) | 69 (22.3) | 0.4 (0.1-1.2) | 0.1 |
| CC | CC | 10 (19.6) | 43 (13.9) | 0.8 (0.3-2.3) | 0.7 |
| CT | AA | 10 (19.6) | 66 (21.3) | 0.5 (0.2-1.2) | 0.2 |
| CT | CC | - | - | - |  |
| TT | AA | 7 (13.7) | 41 (13.2) | 0.6 (0.2-1.9) | 0.4 |
| TT | AC | - | - | - |  |

**p-values were all >0.05*
